# Origin of enhanced chemical precompression in cerium hydride $\hbox {CeH}_{{9}}$

**DOI:** 10.1038/s41598-020-73665-1

**Published:** 2020-10-09

**Authors:** Hyunsoo Jeon, Chongze Wang, Seho Yi, Jun-Hyung Cho

**Affiliations:** Department of Physics, Research Institute for Natural Science, and Institute for High Pressure at Hanyang University, Hanyang University, 222 Wangsimni-ro, Seongdong-ku, Seoul, 04763 Republic of Korea

**Keywords:** Superconducting properties and materials, Chemical physics

## Abstract

The rare-earth metal hydrides with clathrate structures have been highly attractive because of their promising high-$T_{\rm{c}}$ superconductivity at high pressure. Recently, cerium hydride $\hbox {CeH}_9$ composed of Ce-encapsulated clathrate H cages was synthesized at much lower pressures of 80–100 GPa, compared to other experimentally synthesized rare-earth hydrides such as $\hbox {LaH}_{{10}}$ and $\hbox {YH}_6$. Based on density-functional theory calculations, we find that the Ce 5*p* semicore and 4*f*/5*d* valence states strongly hybridize with the H 1*s* state, while a transfer of electrons occurs from Ce to H atoms. Further, we reveal that the delocalized nature of Ce 4*f* electrons plays an important role in the chemical precompression of clathrate H cages. Our findings not only suggest that the bonding nature between the Ce atoms and H cages is characterized as a mixture of ionic and covalent, but also have important implications for understanding the origin of enhanced chemical precompression that results in the lower pressures required for the synthesis of $\hbox {CeH}_9$.

## Introduction

In recent years, rare-earth metal hydrides have attracted much attention due to the possibility of their realization of room-temperature superconductivity (SC)^1–8^. First-principles density-functional theory (DFT) calculations together with the Migdal–Eliashberg formalism have predicted that rare-earth metal hydrides such as yttrium, lanthanum, cerium hydrides host high-$T_{\rm{c}}$ SC at megabar pressures^9,10^, the origin of which is based on phonon-mediated electron pairing^11^. Subsequently, such a conventional SC of $\hbox {LaH}_{{10}}$ was experimentally observed with a superconducting transition temperature $T_{\rm{c}}$ of 250–260 K at a pressure of ${\sim }$170 GPa^3,4^. This $T_{\rm{c}}$ record of $\hbox {LaH}_{{10}}$ has been the highest temperature so far among experimentally available superconducting materials including cuprates^12,13^ and Fe-based superconductors^14,15^. Therefore, the experimental realization of room-temperature SC in $\hbox {LaH}_{{10}}$ has stimulated interests of the high-$T_{\rm{c}}$ community towards compressed metal hydrides under high pressure^16–23^.

**Figure 1:**
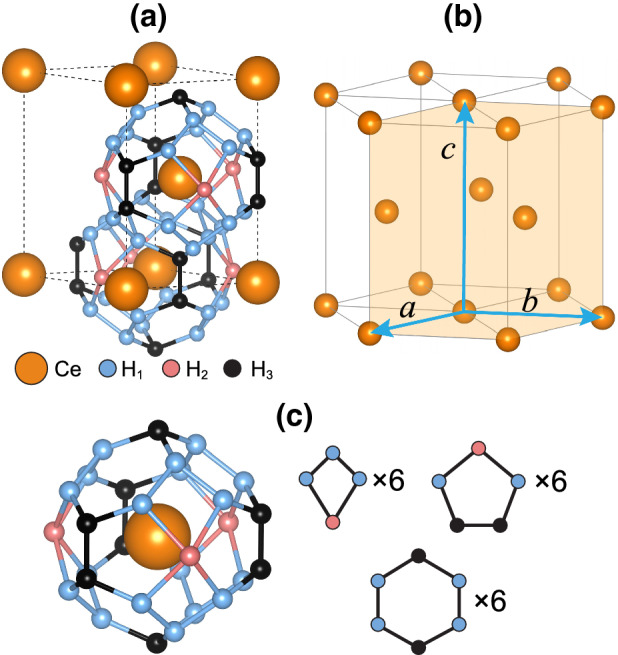
**(a)** Optimized structure of $\hbox {CeH}_{{9}}$ at 100 GPa and **(b)** hexagonal-close-packed (hcp) lattice of Ce atoms. Three different species of H atoms, $\hbox {H}_{{1}}$, $\hbox {H}_{{2}}$, and $\hbox {H}_{{3}}$, exist in $\hbox {H}_{{29}}$ cage. The isolated $\hbox {H}_{{29}}$ cage surrounding a Ce atom is displayed in **(c)**, together with its constituent parts, i.e., six tetragon rings, six pentagon rings, and six hexagon rings.

However, since the synthesis of $\hbox {LaH}_{{10}}$ was performed at ${\sim }$170 GPa^3,4^, it has been quite demanding to discover H-rich rare-earth hydrides synthesized at a moderate pressure below ${\sim }$100 GPa, which is easily and routinely achievable in the diamond anvil cell (DAC)^24,25^. Motivated by the first theoretical prediction^9^ of cerium hydride $\hbox {CeH}_9$ with a clathrate hydrogen cage structure, two experimental groups^5,6^ achieved its successful synthesis at a lower pressure of 80–100 GPa. X-ray diffraction measurements and DFT calculations^5,6^ confirmed the previously predicted^9^ crystal structure of $\hbox {CeH}_9$, which adopts a hexagonal clathrate structure with the space group P6$_3$/*mmc*. Here, each Ce atom is surrounded by the $\hbox {H}_{{29}}$ cage consisting of 29 H atoms (see Fig. 1a). It is remarkable that the H–H bond lengths in $\hbox {CeH}_9$ are close to those of solid metallic hydrogen that can be produced at high pressure above 400 GPa^26–29^. Therefore, the discovery of $\hbox {CeH}_9$ having clathrate hydrogen networks suggests that the metallic state of solid hydrogen can be attained at relatively lower pressures by using binary hydrides with *f*-electron metals. It is noteworthy that the sizable reduction of H–H bond lengths in $\hbox {CeH}_9$ reflects the presence of a larger chemical precompression^30–32^ compared to other rare-earth metal hydrides such as $\hbox {LaH}_{{10}}$ and $\hbox {YH}_{{10}}$^9,10,33–37^. However, the underlying mechanism of how the pressure required for the synthesis of $\hbox {CeH}_9$ is much reduced is yet to be identified.

In this paper, we investigate the electronic structure and bonding properties of $\hbox {CeH}_9$ at high pressure using first-principles DFT calculations with the inclusion of Hubbard on-site Coulomb interaction. The calculated band structure of $\hbox {CeH}_9$ shows a strong hybridization of the Ce 5*p* semicore and 4*f*/5*d* valence states with the H 1*s* state. We reveal that the delocalized nature of Ce 4*f* electrons contributes to yield a much larger chemical precompression of clathrate $\hbox {H}_{{29}}$ cages along the *c* axis than in the *a*–*b* plane. Despite a strong hybridization between the Ce- and H-derived electronic states, our Bader charge analysis shows a charge transfer from Ce to H atoms, thereby suggesting that the bonding nature between the Ce atoms and $\hbox {H}_{{29}}$ cages features the mixed ionic and covalent bonding. The present results provide new insight into understanding the underlying mechanism of the chemical precompression that requires relatively lower pressures for the synthesis of $\hbox {CeH}_9$^5,6^, compared to other experimentally synthesized rare-earth hydrides $\hbox {LaH}_{{10}}$^3,4^ and $\hbox {YH}_{{6}}$^7,8^.

## Results and discussion

**Figure 2:**
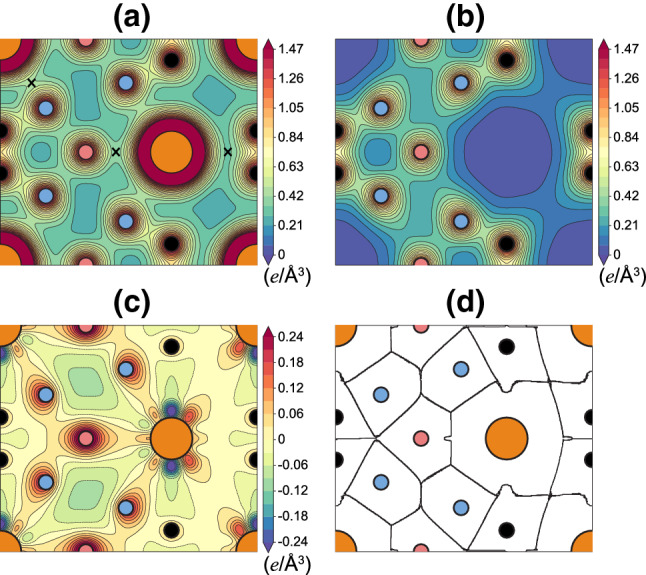
Calculated total charge density ${\rho }_{\rm{tot}}$ of **(a)** $\hbox {CeH}_9$ and **(b)** isolated $\hbox {H}_{{29}}$ cages. The saddle points of charge densities between Ce and $\hbox {H}_1$/$\hbox {H}_2$/$\hbox {H}_3$ atoms are marked “${\times }$” in **(a)**. The charge densities in **(a)** and **(b)** are plotted on the (110) plane with the contour spacings of 0.07 *e*/Å$^3$. The charge density difference ${\Delta }{\rho }$ (defined in the text) is displayed in **(c)**, with the contour spacing of ${\pm }$0.03 *e*/Å$^3$. The Bader basins of Ce and H atoms are displayed in **(d)**.

**Figure 3:**
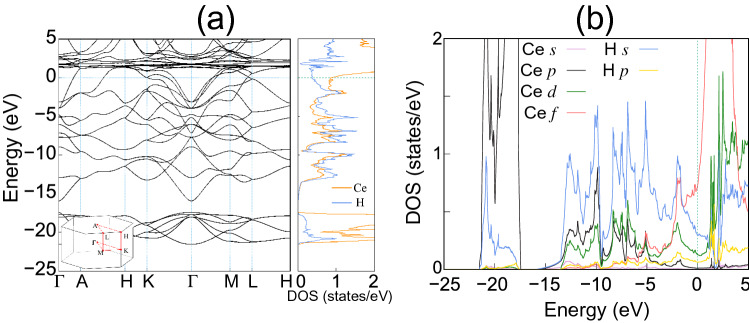
**(a)** Calculated band structure of $\hbox {CeH}_9$ together with the LDOS for Ce and H atoms. The energy zero represents $E_{\rm{F}}$. The PDOS of $\hbox {CeH}_9$ is also given in **(b)**.

We begin by optimizing the structure of $\hbox {CeH}_9$ using the PBE + U calculation. Figure 1a shows the optimized structure of $\hbox {CeH}_9$ at a pressure of 100 GPa, which is the same pressure employed in previous DAC experiments^5,6^. Here, Ce atoms form a hcp lattice (see Fig. 1b) with the lattice constants $a = b = 3.717$ Å and $c = 5.666$ Å, in good agreement with the experimental^5,6^ data of $a = b = 3.66$ Å and $c = 5.58$ Å. Meanwhile, the $\hbox {H}_{{29}}$ cage surrounding a Ce atom is constituted by six tetragon rings, six pentagon rings, and six hexagon rings (see Fig. 1c). Note that there are three species of H atoms [termed $\hbox {H}_1$, $\hbox {H}_2$, and $\hbox {H}_3$ in Fig. 1a composing the $\hbox {H}_{{29}}$ cage. We find that the $\hbox {H}_{1}-\,\hbox {H}_1$, $\hbox {H}_{1}-\,\hbox {H}_2$, $\hbox {H}_{1}-\,\hbox {H}_3$, and $\hbox {H}_{3}-\,\hbox {H}_3$ bond lengths are 1.190, 1.486, 1.275, and 1.065 Å, respectively. These H–H bond lengths in $\hbox {CeH}_9$ are close to those (0.98 and 1.21 Å) predicted from metallic hydrogen at ${\sim }$500 GPa^28^. It is thus likely that the synthesized^5,6^ binary compound $\hbox {CeH}_9$ with the clathrate $\hbox {H}_{{29}}$ cages is able to generate H networks comparable to metallic hydrogen even at a low pressure of 100 GPa.

Figure 2a shows the calculated total charge density ${\rho }_{\rm{tot}}$ of $\hbox {CeH}_9$. It is seen that H atoms in the $\hbox {H}_{{29}}$ cage are bonded to each other with covalent-like bonding. Here, the charge densities of H–H and Ce–H bonds exhibit the saddle-point characters at their midpoints, similar to that of the C–C covalent bond in diamond^38^. As shown in Fig. S1 in the Supplementary information, the calculated electron localization function also shows the covalent-like H–H and Ce–H bonds. The charge densities at the midpoints of the $\hbox {H}_{1}-\,\hbox {H}_2$, $\hbox {H}_{1}-\,\hbox {H}_3$, and $\hbox {H}_{3}-\,\hbox {H}_3$ bonds are 0.39, 0.56, and 0.85 *e*/Å$^3$, respectively. These values in $\hbox {CeH}_9$ are larger than the corresponding ones (0.32, 0.45, and 0.76 *e*/Å$^3$ in Fig. 2b) obtained from the isolated $\hbox {H}_{{29}}$ cages whose structure is taken from the optimized structure of $\hbox {CeH}_9$. This result implies that the H–H covalent bonds in $\hbox {CeH}_9$ are strengthened by a charge transfer from Ce to H atoms. Interestingly, the electrical charges of Ce and H atoms are connected to each other, reflecting a covalent-like bonding character. It is noteworthy that the charge densities at the points marked “${\times }$” (in Fig. 2a) between Ce and $\hbox {H}_1$/$\hbox {H}_2$/$\hbox {H}_3$ atoms are close to that at the midpoint of the $\hbox {H}_{1}-\,\hbox {H}_2$ bond. This covalent nature of Ce–H bonds is attributed to a strong hybridization between the Ce and H electronic states, as discussed below.

**Figure 4:**
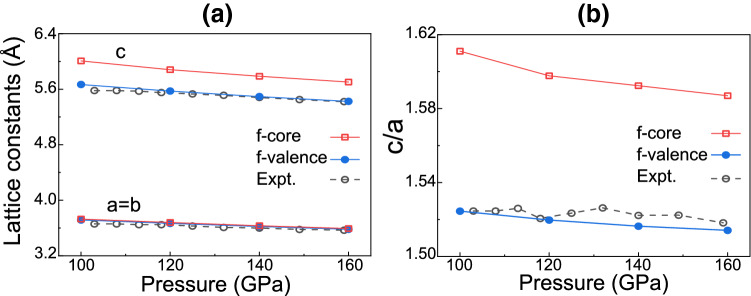
**(a)** Calculated lattice constants of $\hbox {CeH}_9$ as a function of pressure using the *f*-valence scheme, in comparison with those obtained using the *f*-core scheme and experiment^5^. The resulting *c*/*a* ratios as a function of pressure are also given in **(b)**.

To examine the charge transfer between Ce to H atoms, we calculate the charge density difference, defined as ${\Delta }{\rho }$ = ${\rho }_{\rm{tot}}$ − ${\rho }_{\rm{Ce}}$ − ${\rho }_{\rm{H}}$, where ${\rho }_{\rm{Ce}}$ and ${\rho }_{\rm{H}}$ represent the charge densities of the isolated Ce lattice (Fig. 1b) and the isolated $\hbox {H}_{{29}}$ cages (Fig. 2b), respectively. As shown in Fig. 2c, ${\Delta }{\rho }$ illustrates how electronic charge is transferred from Ce to H atoms. It is seen that the charge transfer occurs mostly from Ce to $\hbox {H}_1$ and $\hbox {H}_2$. Meanwhile, the charge transfer from Ce to $\hbox {H}_3$ is minor. We further calculate the Bader charges^39^ of $\hbox {CeH}_9$ to estimate the number of transferred electrons between Ce and H atoms. Figure 2d shows the Bader basins of the constituent atoms, obtained from the gradient of ${\rho }_{\rm{tot}}$^39^. We find that the calculated Bader charges of Ce, $\hbox {H}_1$, $\hbox {H}_2$, and $\hbox {H}_3$ basins are $- 9.47 e$ (including the $5 s^2 5 p^6$ semicore electrons), $-1.34 e$, $-1.31 e$, and $-1.09 e$, respectively. Thus, we can say that Ce atoms lose electrons of 2.53*e* per atom, while $\hbox {H}_1$, $\hbox {H}_2$, and $\hbox {H}_3$ atoms gain electrons of 0.34*e*, 0.31*e*, 0.09*e* per atom, respectively.

In Fig. 3a, we display the calculated band structure of $\hbox {CeH}_9$, together with the local density of states (LDOS) for Ce and H atoms. The narrow bands located at ${\sim }$2 eV above $E_{\rm{F}}$ originate from Ce 4*f* electrons, while those located at around −20 eV below $E_{\rm{F}}$ are associated with Ce 5*p* semicore electrons. It is noticeable that the LDOS shape of Ce atoms is very similar to that of H atoms in the energy range between −15 eV and $E_{\rm{F}}$, indicating a strong hybridization between Ce and H electronic states. In order to resolve the orbital characters of electronic states, we plot the partial density of states (PDOS) projected onto the Ce 5*p*-semicore and 4*f*/5*d*-valence states and the H 1*s* state in Fig. 3b. We find that the Ce 5*p*-semicore states are extended upward to reach up to $E_{\rm{F}}$, while the 4*f*- and 5*d*-valence states are distributed downward to about −10 and −13 eV below $E_{\rm{F}}$, respectively. Hence, these delocalized semicore and valence states hybridize well with the H 1*s* state. Such a strong hybridization between Ce and H electrons is likely associated with the Ce-encapsulated spherical H-cage structure of $\hbox {CeH}_9$. Consequently, the electron charges of Ce and H atoms show covalent characteristics between the Ce and $\hbox {H}_1$/$\hbox {H}_2$/$\hbox {H}_3$ atoms (see the “${\times }$” points in Fig. 2a). Based on this covalent feature of the Ce–H bonds and the charge transfer from Ce to H atoms, we can say that the bonding nature between the Ce atoms to $\hbox {H}_{{29}}$ cages is characterized as a mixture of ionic and covalent.

As mentioned above, the synthesis of $\hbox {CeH}_9$ requires much lower pressures 80–100 GPa^5,6^ compared to that (${\sim }$170 GPa) for the synthesis of $\hbox {LaH}_{{10}}$^3,4^, indicating the variation of chemical precompression with respect to the occupation of *f* electrons. Here, we note that Ce atom with the electron configuration [Xe]4$f^1$5$d^1$6$s^2$ has one 4*f* electron, while La atom with [Xe]5$d^1$6$s^2$ represents no occupation of *f* orbitals. It is thus expected that the lower synthesis pressure of $\hbox {CeH}_9$ would be caused by the presence of the delocalized Ce 4*f* states (see Fig. 3b). To confirm how the delocalized nature of Ce 4*f* electrons contributes to the chemical precompression of $\hbox {H}_{{29}}$ cages, we optimize the structure of $\hbox {CeH}_9$ as a function of pressure using the *f*-core scheme, where Ce 4*f* electrons are considered as core electrons. Therefore, the interactions of 4*f* electrons with valence electrons are completely removed to simulate the localized nature of 4*f* electrons^40^. The band structure and PDOS of $\hbox {CeH}_9$ calculated using the *f*-core scheme are displayed in Fig. S2. We find that the band dispersions of the Ce 5*d* and H 1*s* states change largely around $E_{\rm{F}}$ because their hybridizations with the Ce 4*f* states are avoided in the *f*-core scheme. In Fig. 4a, the lattice parameters computed using the *f*-core scheme are compared with those of the *f*-valence scheme as well as the experimental data^5^. We find that in the pressure range between 100 and 160 GPa, both the *f*-core and *f*-valence schemes predict similar values for *a* and *b*, close to the experimental values^5^. However, the *f*-core scheme predicts larger *c* values than the *f*-valence scheme and experiment^5^ by ${\sim }$6% in the same pressure range. As a result, in contrast to both the *f*-valence scheme and experiment^5^, the *f*-core scheme gives relatively larger values of the *c*/*a* ratio between 100 and 160 GPa (see Fig. 4b). These results indicate that the delocalized nature of Ce 4*f* electrons plays an important role in determining the chemical precompression along the *c* axis, while it hardly affects the chemical precompression in the *a*–*b* plane.

In order to check whether the localized/delocalized nature of Ce 4*f* electrons influences the dynamical stability of $\hbox {CeH}_9$, we calculate the phonon spectrum at 100 GPa using both the *f*-core and *f*-valence schemes. The calculated phonon spectrum of the *f*-core scheme exhibits imaginary frequencies in the whole Brillouin zone [see Fig. S3(a) in the Supplementary information], indicating that $\hbox {CeH}_9$ is dynamically unstable. On the other hand, the *f*-valence scheme shows that $\hbox {CeH}_9$ is dynamically stable without any imaginary-frequency phonon mode [see Fig. S3(b)]. Therefore, we can say that the delocalized nature of Ce 4*f* electrons is necessary for stabilizing the clathrate structure of $\hbox {CeH}_9$.

## Conclusion

Our first-principles DFT + U calculations for $\hbox {CeH}_{{9}}$ have shown that (i) the Ce 5*p* semicore and 4*f*/5*d* valence states strongly hybridize with the H 1*s* state, (ii) the charge transfer occurs mostly from Ce to $\hbox {H}_1$ and $\hbox {H}_2$ atoms, and (iii) the delocalized nature of Ce 4*f* electrons is an essential ingredient in the chemical precompression of clathrate $\hbox {H}_{{29}}$ cages. The present results not only suggest that the bonding nature between the Ce atoms and H cages is characterized as a mixture of ionic and covalent, but also provide an explanation for the enhanced chemical precompression in $\hbox {CeH}_9$. We thus proposed that the large chemical precompression of H-rich clathrate structures can be attained in rare-earth hydrides with delocalized 4*f* electrons. It is noteworthy that, according to DFT calculations, $\hbox {PrH}_9$^16^ with clathrate H cages begins to destabilize at a pressure below about ${\sim }$100 GPa, which is relatively lower than that (226 GPa) of $\hbox {YH}_{{10}}$^36^. These different destabilization pressures of $\hbox {PrH}_9$ and $\hbox {YH}_{{10}}$ are likely to reflect the variation of chemical precompression, due to the influence of delocalized *f* electrons. Indeed, $\hbox {PrH}_9$ was experimentally synthesized at a pressure of ${\sim }$100 GPa^16^. We also note that $\hbox {YH}_6$ containing no *f* electrons begins to destabilize at a pressure below ${\sim }$72 GPa, which is much lower than that (${\sim }$226 GPa) of $\hbox {YH}_{{10}}$^36^. Here, the different stabilization pressures of $\hbox {YH}_6$ and $\hbox {YH}_{{10}}$ were explained^36^ in terms of the size of Y atom: i.e., $\hbox {YH}_{{10}}$ having denser, larger hydrogen cages with shorter H–H distances requires a higher stabilization pressure.

## Methods

Our DFT calculations were performed using the Vienna ab initio simulation package with the projector-augmented wave method^41–43^. For the exchange-correlation energy, we employed the generalized-gradient approximation functional of Perdew–Burke–Ernzerhof (PBE)^44^. The 5$s^2$5$p^6$ semicore electrons of Ce atom were included in the electronic-structure calculations. For Ce 4*f* electrons, we considered the effective on-site Coulomb interaction of $\hbox {U}_{\rm{eff}}$(=U$-J$) = 4 eV, where the Hubbard parameter U is 4.5 eV and the exchange interaction parameter *J* is 0.5 eV^5^. A plane-wave basis was used with a kinetic energy cutoff of 1000 eV. The $\mathbf{k}$-space integration was done with 12${\times }$12${\times }$8 *k* points for the structure optimization and 24${\times }$24${\times }$16 *k* points for the DOS calculation. All atoms were allowed to relax along the calculated forces until all the residual force components were less than 0.005 eV/Å. Phonon calculations were preformed by a finite displacement method with the PHONOPY code^45^.

## Supplementary information

Supplementary information.
