# Supplementary material for: Origin of enhanced chemical precompression in cerium hydride CeH9
Source: Sci Rep. 2020 Oct 9;10:16878. doi: 10.1038/s41598-020-73665-1 (PMC7547066; doi:10.1038/s41598-020-73665-1)
Supplement: Supplementary file 1 — Supplementary information. [file 41598_2020_73665_MOESM1_ESM.pdf]

Supplementary information for “Origin of enhanced chemical precompression in cerium hydride CeH_9”

Hyunsoo Jeon, Chongze Wang, Seho Yi, Jun-Hyung Cho*

Department of Physics, Research Institute for Natural Science, and Institute for High Pressure at Hanyang University, Hanyang University, 222 Wangsimni-ro, Seongdong-Ku, Seoul 04763, Republic of Korea

*Corresponding author: chojh@hanyang.ac.kr

H. J., C. W., and S. Y. contributed equally to this work.

1. Electron localization function of CeH_9

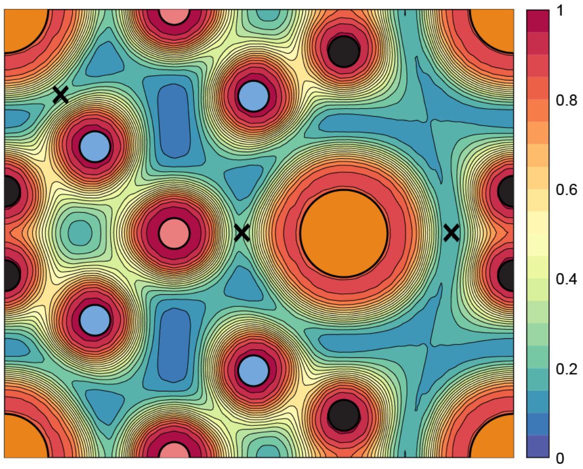

Figure S1. Electron localization function (ELF) of CeH_9[1-3]. The first line is drawn at 0.05 and the contour spacing is 0.05. Low (high) values of ELF are shown in blue (red) color.

2. Band structure and PDOS of CeH_9 in the *f*-core scheme

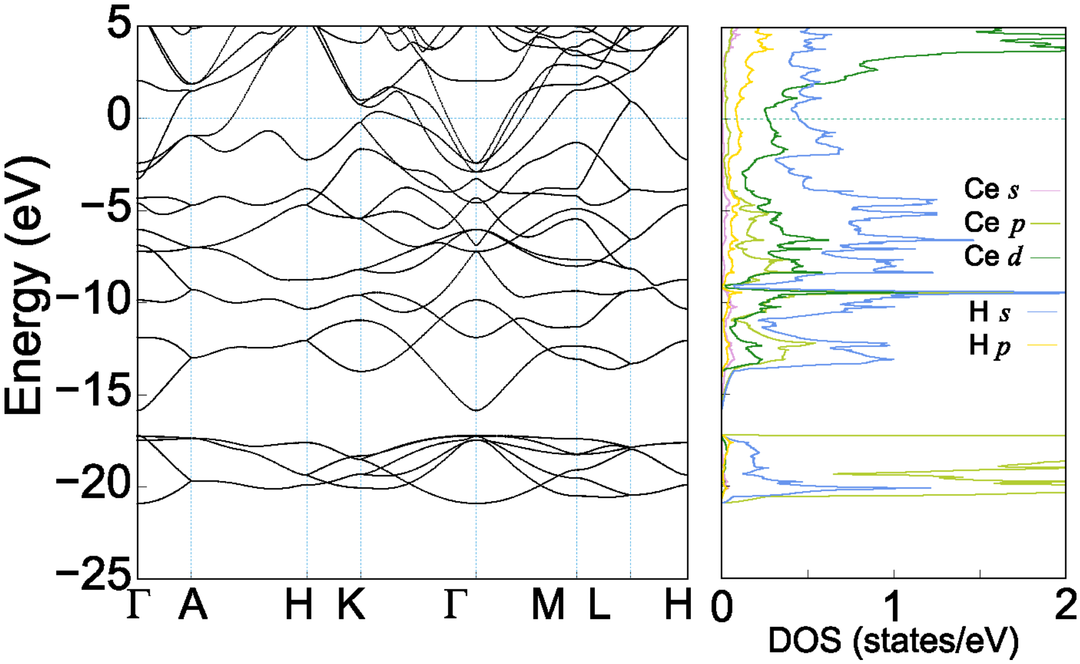

Figure S2. Band structure and PDOS of CeH_9 at 100 GPa, calculated using the *f*-core scheme. Here, we use a special pseudopotential of Ce, in which *f* electrons are kept frozen in the core.

3. Phonon spectra and DOS of CeH_9 in the *f*-core and *f*-valence schemes

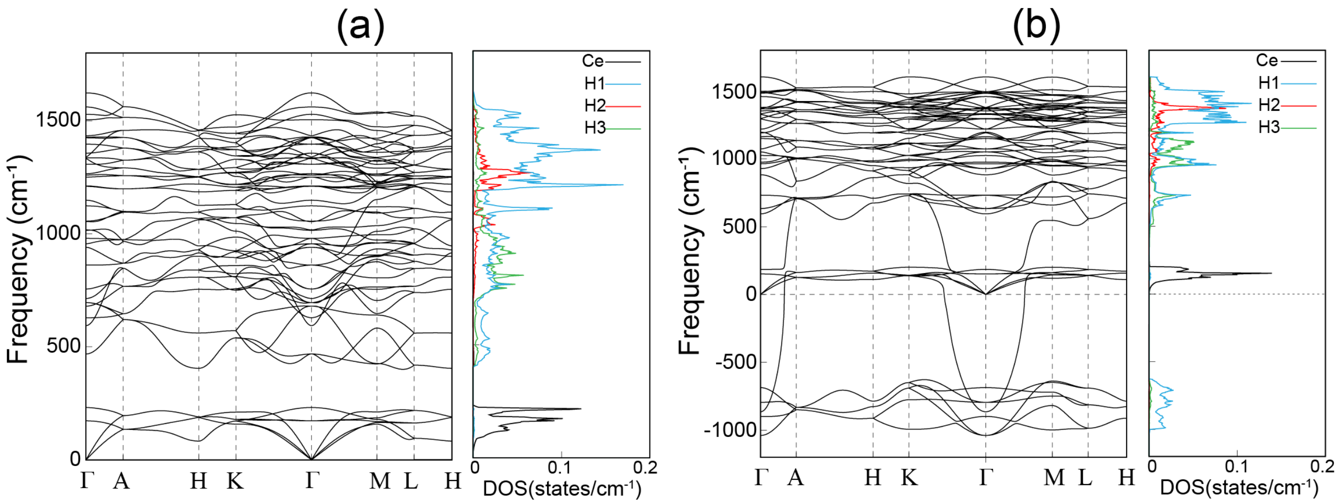

Figure S3. Phonon spectrum and DOS of CeH_9 at 100 GPa, calculated using the (a) *f*-core (b) *f*-valence schemes. The phonon DOS is projected onto Ce, H_1, H_2, and H_3 atoms. Here, the phonon spectra and DOS are obtained using the finite displacement method implemented in PHONOPY package, where the forces are calculated using the VASP code with a 2 × 2 × 2 supercell.

References

[1] Becke, A. D. & Edgecombe, K. E. A simple measure of electron localization in atomic and molecular systems. *J. Chem. Phys.* **92**, 5397 (1990).

[2] Savin, A. *et al.* Electron Localization in Solid-State Structures of the Elements: the Diamond Structure. *Angew. Chem. Int. Ed. Engl.* **31**, 187 (1992).

[3] Silvi, B. & Savin, A. Classification of chemical bonds based on topological analysis of electron localization functions. *Nature* **371**, 683-686 (1994).
